# Global Regulatory Mandates as Drivers for Advanced Chemical Analysis in Food Safety

**DOI:** 10.3390/foods15081454

**Published:** 2026-04-21

**Authors:** Lin Guo, Xiaoxiao Dong, Heng Zhou, Zilong Liu, Xingchuang Xiong

**Affiliations:** 1 Center for Metrology Scientific Data, National Institute of Metrology, Beijing 100029, China; guolin@tju.edu.cn (L.G.); dongxx@nim.ac.cn (X.D.); zhouheng@nim.ac.cn (H.Z.); liuzl@nim.ac.cn (Z.L.); 2 National Metrology Data Center, Beijing 100029, China; 3 Key Laboratory of Metrology Digitalization and Digital Metrology, State Administration for Market Regulation, Beijing 100029, China

**Keywords:** food safety regulation, chemical analysis, Maximum Residue Limits (MRL), method validation, LC-MS/MS, regulatory heterogeneity, Codex Alimentarius, ISO/IEC 17025, risk assessment, high-resolution mass spectrometry

## Abstract

The globalization of the food supply chain presents complex challenges for safety assurance within a highly fragmented regulatory landscape. This review synthesizes the frameworks of eight influential jurisdictions—including the European Union (EU), the United States, China, and Codex Alimentarius—to evaluate how legal mandates function as regulatory drivers that guide the evolution of analytical chemistry. By examining legislation on Maximum Residue Limits (MRLs), positive list systems, and method validation guidelines (e.g., SANTE), we demonstrate that strict preventive controls have established chromatography coupled with tandem mass spectrometry (LC/GC-MS/MS) as the universal standard for multi-residue screening. We show that global regulatory fragmentation is not merely an administrative artifact, but is rooted in divergent toxicological philosophies and localized dietary exposure models. This regulatory heterogeneity requires analytical laboratories to adopt a posture of “defensive technological redundancy,” forcing them to continuously optimize targeted methods against the strictest global default limits (e.g., 0.01 mg/kg). We establish that this continuous methodological escalation for ultra-trace quantification has reached practical and operational limits. Consequently, we conclude that the future of food safety testing must transition from static target-list compliance toward adaptable, non-targeted chemical profiling using High-Resolution Mass Spectrometry (HRMS), enabling laboratories to proactively address emerging contaminants, food fraud, and the complexities of modern food matrices.

## 1. Introduction

### 1.1. Global Regulatory Context and Aims of the Review

The global food supply chain constitutes a vast and intricate network, with trade in food and agricultural products expanding rapidly [1,2]. In 2020, the European Union (EU), the United States, and China were the leading importers of key commodities, with a combined import value exceeding $100 billion, while major exporters included the EU, Brazil, the United States, and New Zealand [3]. This substantial volume of cross-border trade highlights the economic significance of the global food system. However, this interconnectedness also presents significant challenges for ensuring food safety.

This global landscape operates within a complex and fragmented regulatory environment, where the laws and standards governing food safety are frequently inconsistent across different nations [1]. This regulatory heterogeneity functions as a technical barrier to trade, potentially generating disputes and compliance challenges for producers exporting to multiple markets [2]. For instance, the EU maintains one of the most comprehensive and stringent regulatory systems worldwide, employing a “farm-to-fork” approach that often sets standards stricter than those of other regions [4,5]. In contrast, other major economies, such as China, are in the process of integrating thousands of overlapping and sometimes contradictory standards into a single, unified National Food Safety Standards (NFSS) framework to enhance coherence and enforcement [6,7,8]. Concurrently, regional blocs like Australia and New Zealand (through FSANZ) and the United States and Canada (through acts like FSMA and SFCA) are pursuing cooperative agreements and similar approaches to harmonize regulations and facilitate trade [1,9]. International bodies, most notably the Codex Alimentarius Commission (CAC), serve as a global reference point, developing standards and guidelines that are recognized by the World Trade Organization (WTO) to promote fair trade and protect consumer health [10,11,12]. Disparities in Maximum Residue Limits (MRLs) for contaminants, pesticides, and veterinary drugs across markets like the EU, the United States, and Japan [2] create significant compliance bottlenecks for international trade.

Among these diverse chemical hazards, pesticide residues exemplify the most representative paradigm of this dynamic between regulatory mandates and analytical evolution. Plant protection products are integrated into modern agricultural practices to safeguard the yield and commercial quality of cereals, fruits, and vegetables. These substances remain essential for controlling pests during cultivation, transport, and storage, thereby mitigating post-harvest deterioration and the emergence of undesirable mycotoxins [13,14]. However, the declining availability of approved active substances, particularly for minor crops, increasingly restricts and complicates effective agricultural protection strategies [15]. Concurrently, the extensive application of these chemicals introduces systemic food safety hazards. From a toxicological perspective, dietary exposure to pesticide residues presents measurable acute and chronic human health risks, including established mechanisms of neurotoxicity, endocrine disruption, and carcinogenicity [13,15]. Furthermore, the co-occurrence of multiple residues generates amplified cumulative and synergistic health risks, posing a disproportionate toxicological burden on vulnerable demographics, particularly children [14].

To mitigate these documented toxicological threats, regulatory frameworks enforce increasingly stringent exposure thresholds. Authorities systematically withdraw approvals for high-risk active substances and continuously lower MRLs [13]. A specific manifestation of this regulatory tightening is the enforcement against chlorpyrifos. Despite its unapproved status, it frequently accounts for significant MRL violations, with exceedances reaching up to 420% above the legal limit in commodities such as oranges [14]. Consequently, regions like the EU restrict the MRL for such unauthorized substances to the statutory default limit of 0.01 mg/kg [15]. This statutory compression of legal thresholds challenges the current technological capabilities of modern mass spectrometry, driving continuous instrumental optimization to achieve ultra-trace LOQs. Furthermore, the extensive number of regulated pesticides across different jurisdictions necessitates the use of robust multi-residue methods (MRMs), which rely on advanced chromatographic and mass spectrometric techniques to simultaneously screen for a wide array of compounds [4,16]. Moreover, international guidelines, such as those from the CAC and the International Organization for Standardization (ISO), stipulate the performance requirements for these analytical methods. They establish criteria for method validation, including parameters such as accuracy, precision, and repeatability, to ensure that analytical data are scientifically valid, reliable, and defensible across different legal and trade frameworks [17,18,19].

**Figure 1:**
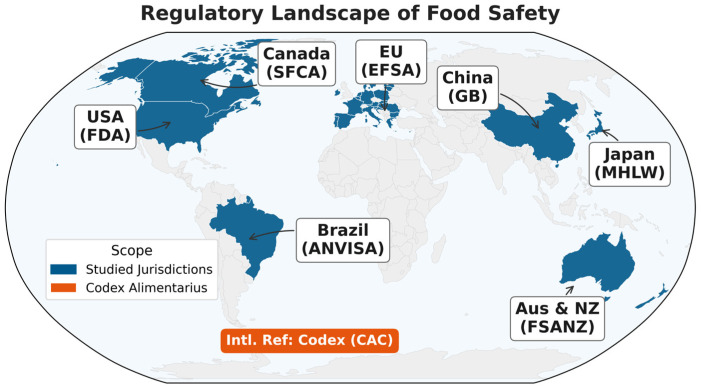
Geographic scope of the regulatory frameworks and international standards examined in this review. The map highlights the seven key national and regional jurisdictions (blue) and the international standard-setting body, Codex Alimentarius (orange), selected for this analysis. These entities were chosen based on their dominant share of global agricultural trade and their pivotal role in defining benchmark analytical requirements for food safety.

Given that the validation and optimization of any analytical method are dictated by the specific compliance targets of the importing market, navigating global food trade requires a precise understanding of these diverging standards. To bridge the gap between macroscopic regulatory mandates and microscopic analytical execution, this review synthesizes the frameworks of eight influential jurisdictions and organizations: China, the United States, the European Union, Canada, Brazil, Japan, Australia/New Zealand, and the Codex Alimentarius Commission (Figure 1). It is important to note that Figure 1 represents the regulatory scope rather than the structural organization of this review. Instead of providing a region-by-region inventory, we utilize these frameworks as representative case studies. By incorporating key regulatory jurisdictions, major export hubs, strict positive-list enforcers, and the global Codex baseline, this selection captures the full spectrum of modern regulatory paradigms. Collectively, these entities account for the majority of global food trade value; therefore, their requirements offer a comprehensive proxy for demonstrating how regulations function as regulatory drivers for chemical analysis.

Building upon this representative framework, the primary purpose of this study is to analyze the structural impact of these global regulations on laboratory methodology. Specifically, this review aims to: (1) trace how the global legislative shift toward proactive prevention and the establishment of standardized laboratory infrastructures have created a universally rigorous, high-throughput testing mandate; (2) demonstrate that regulatory fragmentation is not merely an administrative artifact, but is rooted in deeper causes—specifically, divergent toxicological philosophies and localized dietary exposure models—which require laboratories to utilize chromatography coupled with tandem mass spectrometry (e.g., LC/GC-MS/MS) to meet diverse compliance limits; and (3) demonstrate that the continuous methodological escalation required for ultra-trace targeted quantification has reached practical and operational limits, necessitating a paradigm shift toward non-targeted High-Resolution Mass Spectrometry (HRMS). Ultimately, this study seeks to prove that navigating the modern food trade requires analytical laboratories to evolve from passive compliance checkpoints into adaptable, data-driven arbiters of global food integrity.

### 1.2. Review Methodology and Scope

#### 1.2.1. Rationale for the Selected Jurisdictions

To ensure an objective and representative scope, the selection of the jurisdictions examined in this review was derived through a data-driven approach that strategically balances universality and uniqueness. The initial filtering was based on total food trade volume. By referencing the Top 10 exporters and importers of food (2022) data published by the World Trade Organization (WTO), we first identified the major economies that dominate both ends of the global supply chain. The European Union, the United States, China, and Canada consistently rank at the top of both global importation and exportation lists (Table 1). Because of their dual-market dominance, the legal limits set by these specific entities act as key technical references for the rest of the world.

To complement this universality with specific regulatory uniqueness, we purposefully selected significant single-directional trade participants. Japan was included as a high-dependency importer (ranking 4th globally at 80 billion USD) to represent jurisdictions that enforce comprehensive Positive List Systems. Conversely, Brazil was selected as a major export-oriented economy (ranking 3rd globally at 132 billion USD) to illustrate how exporting nations adapt to diverse international compliance pressures.

Furthermore, Australia and New Zealand were integrated as a special case study. While Australia is a top-tier exporter, the region’s distinctive feature lies in its bi-national collaborative governance—Food Standards Australia New Zealand (FSANZ)—which provides a distinct model of cross-border risk analysis and standard-setting [9]. Finally, the Codex Alimentarius Commission (CAC) was included not as a sovereign market, but as the essential international baseline against which all aforementioned national frameworks are calibrated [10]. Ultimately, this specific cohort of eight jurisdictions captures the vast majority of international food trade value while representing the full spectrum of modern regulatory philosophies.

#### 1.2.2. Information Acquisition Strategy and Selection Criteria

Unlike traditional systematic reviews that rely exclusively on highly rigid database queries, exploring the intersection of international food safety regulations and analytical chemistry necessitates a more flexible, multi-channel information acquisition strategy. To ensure methodological transparency, the literature and data gathered for this narrative review were systematically sourced across three distinct channels:

Official Legislative and Regulatory Portals: Primary statutory texts, Maximum Residue Limits (MRLs), and risk assessment reports were directly retrieved from the official databases of the selected jurisdictions. Key sources included the Codex Alimentarius official platform, the EUR-Lex database for European Commission regulations, the European Food Safety Authority (EFSA) Journal, and equivalent governmental portals for the FDA (USA) and SAMR (China). (Note: The detailed organizational structures and specific administrative mandates of these agencies are systematically introduced in Section 2 and Section 3).

Technical Standards and Method Validation Guidelines: Official methodological directives governing laboratory compliance were extracted from recognized standard-setting bodies. This included retrieving the SANTE guidelines for pesticide residue analysis, ISO/IEC 17025 documentation, and specific national standard depositories (e.g., the Chinese GB standard series) to trace exact instrumental performance requirements.

Scientific Literature Databases: To capture the physical execution and methodological challenges of these regulations, a targeted search of the Web of Science Core Collection was conducted. The overarching search logic was constructed by intersecting geographic identifiers with specific matrices and analytical techniques (e.g., [Country/Region] AND [Food/Commodity] AND [Detection Method]). This approach efficiently captured how specific regional mandates drive localized technological adaptations.

By definition of the review’s scope, the gathered literature inherently focuses on currently enforced regulatory frameworks and contemporary peer-reviewed analytical methodologies, omitting superseded legal standards or purely clinical toxicological studies that lack direct implications for chemical analysis.

## 2. The Legislative Mandate: Translating Preventive Policy into Analytical Requirements

The global framework for food safety is built upon a foundation of national and international legislation. These legal instruments serve a dual purpose: protecting public health and ensuring fair practices in trade. For the analytical chemist, this legal landscape is essential, as it defines the mandate to test. Food safety laws translate public health goals into specific, measurable, and enforceable requirements, thereby establishing the demand for robust chemical analysis. The results of such analyses must be legally defensible, a requirement that necessitates validated methods, accredited laboratories, and a deep understanding of the regulatory context in which they operate [17,21].

### 2.1. The Evolution from Reaction to Prevention

A significant global trend in food safety legislation has been a philosophical shift from a reactive to a preventive model [1]. Historically, food safety systems were often responsive, with regulations being developed or strengthened following major food safety incidents, such as China’s 2009 Food Safety Law, which was enacted in the aftermath of the 2008 melamine scandal [17,22].

More recent legislative reforms, however, emphasize proactive prevention. The United States’ Food Safety Modernization Act (FSMA), signed into law in 2011, represents the most comprehensive reform of its food safety laws in over 70 years, with a core aim of shifting the focus from responding to contamination to preventing it [1,23]. Central to this is the Hazard Analysis and Risk-Based Preventive Controls (HARPC) system, which mandates a formalized, written safety plan for food manufacturing and processing facilities [21,24]. Operationally, HARPC requires establishments to prospectively evaluate known or reasonably foreseeable risks. This comprehensive hazard analysis incorporates traditional chemical, physical, and biological contaminants, while explicitly anticipating radiological hazards, natural toxins, unapproved additives, and economically motivated adulteration (EMA) [21]. Once these risks are evaluated, facilities must implement and document risk-based preventive controls designed to significantly mitigate or prevent the occurrence of such hazards before the product advances in the production cycle [25]. This proactive evolution is mirrored globally. In China, the amended 2015 Food Safety Law moved the country towards a more integrated and preventive framework, building on lessons from past incidents [7,22]. Similarly, the European Union codifies this strategy through its General Food Law (Regulation (EC) No 178/2002), establishing the ‘farm-to-fork’ framework [26,27], alongside equivalent updates in Canada (SFCA) and Japan (Food Sanitation Act) [22,28].

This global convergence towards preventive legislation shifts the operational focus of analytical chemistry. While end-product compliance testing remains the final regulatory requirement, the modern laboratory increasingly transitions into a statutory validation tool. Routine analytical tasks are thus progressively integrated earlier into the supply chain—prioritizing early-stage raw material screening and continuous environmental monitoring—to explicitly verify the efficacy of preventive hazard controls at their source. This shift from oversight to foresight creates a legal obligation for verification, which is codified through the specific statutes and international standards examined hereafter.

### 2.2. Key Statutes: Global Convergence and Regional Heterogeneity

While the preventive philosophy defines regulatory intent, its operational enforcement relies on specific national and regional statutes. These primary legal frameworks are increasingly influenced by international standards, most notably those established by the Codex Alimentarius Commission (CAC) [22]. As members of the World Trade Organization (WTO), countries are obligated to ensure their food safety measures are consistent with international standards and based on risk analysis principles to avoid creating unnecessary technical barriers to trade [29]. Consequently, many national regulatory frameworks, such as China’s NFSS, are aligned with Codex guidance [22]. This drive for harmonization extends to major agricultural exporters like Brazil (ANVISA), which synchronizes its residue limits for export commodities, and to bi-national alliances like Australia and New Zealand (FSANZ), which utilize shared risk analysis protocols. However, the WTO framework permits jurisdictions to deviate from Codex standards when scientifically justified by localized risk assessments reflecting specific national food consumption patterns (further detailed in Section 5.2). While this inherent regulatory fragmentation persists, the overarching necessity for harmonization dictates that analytical methods must bridge domestic requirements and global benchmarks to facilitate international trade. [17,30]. Consequently, these foundational legislative frameworks impose stringent technological requirements on analytical facilities. A comparative summary of these key legislative frameworks and their specific implications for chemical analysis is provided in Table 2.

A cross-jurisdictional analysis of the eight legislative frameworks in Table 2 reveals a clear dichotomy in global food safety governance.

The Homogeneity of Intent: The similarity across all eight entities lies in their unified philosophical shift from reactive ex-post enforcement to proactive ex-ante risk prevention. This global consensus indicates that relying solely on end-product testing is insufficient; chemical analysis must be systematically embedded earlier in the supply chain to verify preventive controls at their source.

The Heterogeneity of Execution: However, a distinct heterogeneity emerges in how this preventive philosophy is legally operationalized. Although the ultimate goal of risk mitigation is identical, the regulatory mechanisms diverge into three distinct paradigms: Process-Oriented Prevention (e.g., USA, Canada): Frameworks like FSMA and SFCA mandate the continuous verification of facility-level hazard analysis (HARPC/PCPs), shifting the analytical focus toward environmental monitoring and raw material screening. Threshold-Oriented Prevention (e.g., EU, Japan): In contrast, the General Food Law and Food Sanitation Act leverage the precautionary principle. They implement prevention primarily through exhaustive “positive lists,” utilizing strict default statutory limits (e.g., 0.01 mg/kg) as a regulatory threshold to manage unassessed chemical hazards. System-Integration Prevention (e.g., China, Brazil): These jurisdictions focus on consolidating fragmented historical standards into unified, “strictest supervision” networks, actively harmonizing with Codex (or stricter regional limits) to secure both domestic safety and international market access.

This heterogeneity dictates a practical challenge for analytical chemistry. It indicates that although the global intent is uniformly “preventive,” laboratories operating in the international trade network cannot adopt a singular analytical strategy. They are required to operate a dual-capability framework: simultaneously executing continuous upstream process-verification (to satisfy North American mandates) and maintaining ultra-trace, broad-spectrum threshold-compliance screening (to meet the strict regulatory limits of the EU and Japan).

### 2.3. The Mandate for Verification and Its Analytical Implications

The diverse legislative frameworks summarized in Table 2 converge on a single functional requirement: the rigorous analytical verification of chemical compliance. This preventive mandate is operationally achieved by defining strict legal limits for chemical hazards and stipulating robust monitoring programs to enforce them. Consequently, this legal necessity directly dictates three critical analytical dimensions: baseline sensitivity, procedural scope, and evidentiary defensibility.

#### 2.3.1. Maximum Residue Limits (MRLs) and Method Sensitivity

A primary function of food safety legislation is to establish MRLs for pesticides and veterinary drugs, and Maximum Levels (MLs) for contaminants such as heavy metals and mycotoxins [8,30]. The determination of a numerical limit immediately dictates a critical performance requirement for analytical methods: the Limit of Quantitation (LOQ). The analytical method employed for enforcement must possess an LOQ sufficiently low to reliably determine whether a sample is compliant. For example, the stringent 0.01 mg/kg default MRLs enforced by the EU and Japan [2,36] effectively establish a universal baseline sensitivity target for modern screening methods. Furthermore, modern regulatory frameworks continually push these analytical boundaries even lower. For instance, the European Commission Regulation (EU) 2023/915 establishes stringent maximum levels for heavy metals in infant formulas and baby foods (e.g., 0.01 mg/kg for lead), necessitating the deployment of highly sensitive and validated techniques such as Inductively Coupled Plasma Mass Spectrometry (ICP-MS) [21].

#### 2.3.2. Scope of Regulation and Multi-Residue Methods (MRMs)

The extensive number of regulated compounds makes single-analyte testing impractical for routine monitoring. The EU, for instance, has harmonized MRLs for over 1300 pesticides [36]. To efficiently enforce these regulations, laboratories must employ Multi-Residue Methods (MRMs) capable of screening for hundreds of compounds in a single analysis. This regulatory landscape has been a major driver for the widespread adoption of advanced instrumentation, particularly high-performance chromatographic platforms coupled with tandem mass spectrometry. Legally mandated surveillance would be unfeasible without these integrated high-throughput instrumental systems.

#### 2.3.3. Legal Defensibility and Method Validation

For a test result to be used in a regulatory action—such as a product recall, import rejection, or legal prosecution—it must be “legally defensible.” This requirement dictates that the analytical method be formally validated to demonstrate it is fit for purpose [17]. International guidelines, such as those from Codex and ISO/IEC 17025, specify the parameters for validation, including accuracy (recovery), precision, selectivity, and linearity [37]. These performance characteristics are not merely scientific metrics; they are legal prerequisites. Furthermore, modern legislation increasingly integrates quality assurance by mandating the use of competent testing bodies. The United States FSMA, for example, explicitly requires that food testing be performed by accredited laboratories, formally aligning legal compliance with ISO/IEC 17025 standards [21]. Ultimately, this legal framework ensures that analytical chemistry in food safety is no longer merely an academic exercise, but a mandatory, legally defensible tool for risk management.

In summary, while the global food safety landscape has reached a consensus on the transition from reactive enforcement to proactive prevention, this shared philosophy masks a complex regulatory environment. Although international guidelines converge on a universal baseline of technical requirements—specifically the mandate for ultra-trace sensitivities (LOQs), expansive screening (MRMs), and certified quality assurance (ISO/IEC 17025)—the true complexity lies in the heterogeneous logic used to operationalize these mandates. Ultimately, navigating this regulatory heterogeneity elevates the strategic importance of analytical laboratories. No longer functioning merely as generic endpoints for product clearance, testing institutions have evolved into essential technical entities, bearing the critical responsibility of ensuring compliance across diverse global standards.

## 3. Regulatory and Laboratory Infrastructure for Chemical Analysis

**Figure 2:**
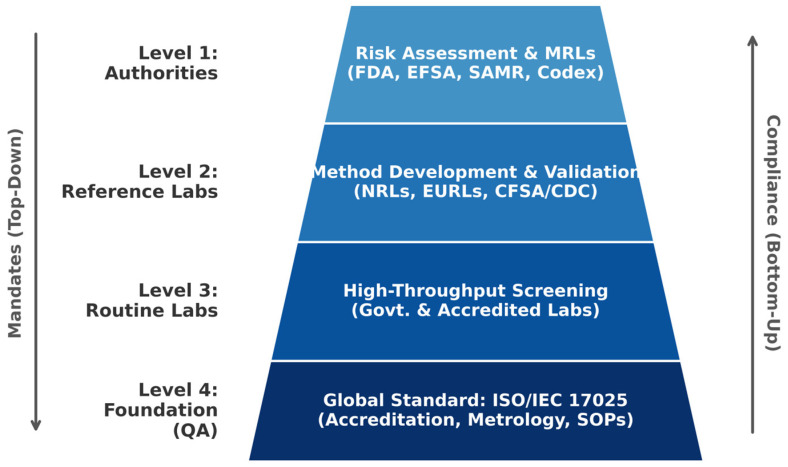
The hierarchical infrastructure of chemical analysis in global food safety systems. The pyramid illustrates the vertical integration of analytical mandates, flowing from regulatory authorities (Level 1) through National Reference Laboratories (Level 2) to frontline routine laboratories (Level 3). The entire structure is underpinned by the universal quality assurance standard, ISO/IEC 17025 (Level 4), which ensures data reliability and international comparability. Arrows indicate the top-down flow of method standardization and the bottom-up flow of compliance data.

The integrity of the global food supply relies on a robust network of regulatory frameworks and the analytical infrastructure designed to enforce them. These systems, which vary by region, dictate the requirements for chemical analysis by establishing legal limits for contaminants and prescribing the methods and quality assurance protocols for their measurement. This section examines the organizational structures and laboratory hierarchies that translate food safety policy into tangible analytical practice, focusing on how governance models, reference laboratories, and quality standards collectively ensure the reliability of data used for food safety control. This multi-layered infrastructure, connecting high-level policy to foundational quality assurance, is illustrated in Figure 2.

### 3.1. Governance Models and Their Analytical Mandates

The architecture of a nation’s food safety governance directly shapes the scope and nature of its chemical testing programs. Broadly, regions employ either multi-agency or centralized models, each with distinct implications for analytical chemistry.

Multi-agency systems, common in large federal nations, distribute responsibilities across several bodies. In the United States, for example, oversight is shared among the Food and Drug Administration (FDA), the Department of Agriculture’s Food Safety and Inspection Service (USDA-FSIS), and the Environmental Protection Agency (EPA) [23]. For analytical chemists, such multi-agency fragmentation often translates to methodological divergence, where distinct agencies may mandate divergent sample preparation protocols and quality assurance criteria for the same analyte. Historically, China’s system was highly fragmented, with roles split among numerous ministries like the Ministry of Health (MoH) and Ministry of Agriculture (MoA) [12]. Such structures can create coordination challenges, which has prompted reforms like China’s move to consolidate enforcement under the State Administration for Market Regulation (SAMR) to improve predictability and efficiency [22,38].

In contrast, other regions utilize more centralized or coordinated models. The European Union (EU) features a highly integrated system where the European Food Safety Authority (EFSA) provides centralized scientific risk assessment, while competent authorities in each Member State conduct official controls [39]. Australia and New Zealand collaborate through Food Standards Australia New Zealand (FSANZ) to develop a unified Food Standards Code, although enforcement in Australia remains distributed across federal, state, and local governments [9].

From an analytical perspective, these governance models are essential because they act as the primary source of the legally binding Maximum Residue Limits (MRLs) discussed in Section 2 [22,40,41]. Whether driven by scientific risk assessments from bodies like EFSA—which inform legislative promulgation by the European Commission—or through consolidated directives from SAMR, these structures define the target lists and regulatory thresholds that the entire laboratory network is required to enforce.

### 3.2. The Role of National Reference Laboratories in Method Standardization

Translating these statutory MRLs into executable laboratory actions requires rigorous method standardization. Consequently, within the food safety infrastructure, a clear hierarchy of quality assurance exists, with National Reference Laboratories (NRLs) positioned at the highest level. These institutions serve as the scientific foundation of the regulatory system, responsible for developing, validating, and disseminating the reference methods used for official control [7,42].

The EU provides a clear example for this hierarchical model with its network of European Union Reference Laboratories (EURLs), each specializing in a specific domain like pesticide residues or mycotoxins. EURLs develop and validate reference methods, which are then transferred to the NRLs within each Member State. These NRLs, in turn, ensure that the official control laboratories in their country can correctly implement these methods [42]. In China, bodies like the National Center for Food Safety Risk Assessment (CFSA) and the Chinese Center for Disease Control and Prevention (CDC) perform analogous functions, providing the core technical expertise for developing the National Food Safety Standards (NFSS), which include a comprehensive set of official testing methods [24]. These methods are published as national standards (e.g., GB, NY/T series) and often specify advanced hyphenated techniques for trace element analysis or tandem mass spectrometry for comprehensive residue screening [43,44].

The work of NRLs is central to modern analytical chemistry. They are tasked with developing and validating robust, fit-for-purpose methods—such as the high-throughput MRMs detailed previously—to ensure they meet stringent regulatory requirements before routine laboratory use. The development process involves rigorous validation to characterize method performance, including parameters like accuracy (recovery), precision (repeatability and reproducibility), specificity, and sensitivity (LOD/LOQ), often following internationally recognized guidelines such as those from the EU (SANTE) or Codex Alimentarius [22].

A second key function of NRLs and EURLs is the organization of Proficiency Testing (PT) schemes [42]. In PT schemes, identical samples are distributed to a network of laboratories for analysis, and their results are compared against a reference value. This process is a key component of external quality control, providing an objective assessment of a laboratory’s competence and the comparability of data across the entire surveillance system. The necessity for ad hoc PT schemes is often triggered by sudden food safety emergencies, requiring the coordination of analytical efforts for novel or unexpected hazards.

### 3.3. Routine Control Laboratories: The Primary Analytical Network

Executing the mandates set by regulators and the methods standardized by NRLs is the primary network of routine control laboratories. This network comprises official government laboratories at national, regional, and local levels, as well as officially recognized or accredited private-sector laboratories. Their primary function is the high-throughput analysis of food samples as part of national monitoring and surveillance programs [12,36].

In the EU, these laboratories are operated by the competent authorities in each Member State and are responsible for implementing both national control plans and the coordinated EU-wide monitoring programs [36,45]. China’s surveillance system relies on a multi-tiered network of provincial and municipal labs that analyze hundreds of thousands of samples annually to enforce its NFSS framework [12]. The U.S. Food Safety Modernization Act (FSMA) similarly mandates that much of the required testing be performed by accredited laboratories, acknowledging the essential role of both public and private labs in the national system [21]. These laboratories must possess highly automated instrumental infrastructure capable of high-throughput screening, alongside the technical expertise to perform official methods reliably and ensure rapid sample turnaround [44,46]. Their performance and the validity of their results are continuously verified through participation in the PT schemes managed by their respective NRLs.

### 3.4. Quality Assurance and ISO/IEC 17025 Accreditation

The entire laboratory infrastructure is supported by a commitment to Quality Assurance (QA), which is benchmarked by the International Organization for Standardization (ISO). Specifically, ISO/IEC 17025, “General requirements for the competence of testing and calibration laboratories,” serves as the international standard for analytical laboratory accreditation.

As established in Section 2, accreditation to ISO/IEC 17025 is increasingly a statutory requirement across major jurisdictions. It provides formal, third-party recognition that a laboratory operates a robust quality management system and is technically competent to generate robust, regulatory-grade data. The importance of this standard is explicitly recognized in regulatory texts, which state that analytical results from different laboratories are only comparable if they are generated under the guidelines of ISO/IEC 17025 [17].

For the analytical chemist, ISO/IEC 17025 translates regulatory requirements into a concrete set of laboratory practices. It mandates documented procedures for method validation, estimation of measurement uncertainty, establishment of metrological traceability, and ongoing quality control, including regular participation in PT schemes. By adhering to this standard, a laboratory ensures that its analytical results are not only accurate and precise but also legally defensible and internationally comparable. This framework of accreditation provides the assurance that the analytical result is reliable across different jurisdictions, thereby enabling a global system of food safety based on shared principles of scientific and technical competence.

Global regulatory systems operate through a consistent hierarchy: governance models establish legal targets, National Reference Laboratories (NRLs) standardize methodologies, and control laboratories conduct routine screening. Supported by the ISO/IEC 17025 standard, this institutional framework provides the necessary foundation to explore how specific regulatory limits dictate analytical method choices and instrumental configurations in the subsequent sections.

## 4. Regulatory Frameworks as the Foundation for Chemical Analysis

Food safety regulations function as more than legal instruments; they serve as foundational specifications that dictate the scope, sensitivity, and methodology of chemical analysis in food safety laboratories. For the analytical chemist, a nation’s or region’s standards system acts as a technical requirement document, defining precisely what to target and the sensitivity required for detection. Maximum Residue Limits (MRLs) for pesticides and veterinary drugs, along with Maximum Levels (MLs) for contaminants, are not merely legal thresholds but translate directly into required analytical performance criteria, specifically the Limit of Quantitation (LOQ) and Limit of Detection (LOD) [47,48]. These regulatory frameworks define the target analyte list, mandate the necessary instrument sensitivity, and drive the evolution of analytical technology toward greater efficiency and precision. This section explores how regulatory systems in key global regions shape the operational landscape of chemical analysis for food safety.

### 4.1. Regulatory Paradigms: Risk-Based vs. Hazard-Based Approaches

The heterogeneity observed in global Maximum Residue Limits (MRLs) originates from divergences in underlying regulatory philosophies, specifically the contrast between hazard-based frameworks and risk-based paradigms. The regulatory architecture of the European Union (EU) increasingly operates on the premise of the precautionary principle and intrinsic hazard identification [49]. This approach dictates that agrochemicals presenting specific hazard profiles—such as mutagenicity, carcinogenicity, reproductive toxicity, or endocrine-disrupting properties—are subjected to strict cut-off criteria, resulting in their systematic non-renewal or prohibition [40,50,51]. In these instances, regulatory decisions are executed independently of actual dietary exposure assessments, as demonstrated by the withdrawal of substances like carbendazim and mancozeb [51]. Furthermore, when scientific uncertainty persists regarding safety thresholds, particularly for vulnerable demographics such as infants, the EU routinely enforces a stringent default MRL of 0.01 mg/kg [45,52]. This hazard-centric model necessitates the development of analytical methodologies with low limits of quantification (LOQs) to verify the absolute minimum presence of unauthorized substances.

This mechanism contrasts with the risk-based methodologies predominantly adopted by the United States and the Codex Alimentarius Commission. These frameworks operate on the premise that the presence of a potentially hazardous substance remains acceptable provided that the estimated dietary exposure does not exceed established toxicological safety thresholds, including the Acceptable Daily Intake (ADI) or Acute Reference Dose (ARfD) [47,53]. By employing quantitative exposure assessments, risk quotient (RQ) calculations, and the “As Low As Reasonably Achievable” (ALARA) principle, these regulatory systems evaluate the probability of harm alongside agricultural requirements and economic viability [8,40,54]. Consequently, substances that face bans in the EU due to inherent hazard characteristics may still receive approved MRLs under Codex or United States regulations if evaluations by bodies like the Joint FAO/WHO Meeting on Pesticide Residues (JMPR) confirm that the exposure quotient is quantitatively acceptable [18].

Ultimately, this difference in approach manifests as regulatory discrepancies across jurisdictions [27,55]. The procedural incompatibility between hazard-based exclusions and risk-based exposure allowances regularly results in diverging residue definitions and conflicting MRL recommendations for identical data sets [56,57]. This ongoing divergence indicates that the global harmonization of international standards, such as Codex CXLs, remains constrained, thereby maintaining the complexity and scope of analytical method validation required for international trade compliance [3,11].

### 4.2. The Analytical Scope: From Negative to Positive List Systems

Regulatory approaches to chemical residues can be broadly categorized into negative and positive list systems, each imposing distinct demands on analytical laboratories. Historically, some systems operated on a negative list basis, specifying only substances that were explicitly prohibited. However, to provide more comprehensive consumer protection, major economic regions have transitioned to a positive list system. This approach, adopted by jurisdictions such as the European Union (EU) and Japan, permits only those substances explicitly listed and for which a specific MRL has been established through risk assessment [2,36].

From an analytical perspective, the shift to a positive list system significantly expands the scope of required testing. Laboratories must be capable of screening for an extensive catalogue of approved compounds. Japan, for instance, introduced its positive list system in 2006, comprehensively reviewing and establishing MRLs for a wide range of pesticides and veterinary drugs [58]. Similarly, the EU’s framework under Regulation (EC) No 396/2005 establishes harmonized MRLs for over 1300 pesticides across 378 food products [45]. While single-residue methods remain a statutory and functional necessity for chemically distinct classes that preclude generic extraction, the extensive list of targets renders single-residue methods impractical, creating a direct regulatory driver for the adoption of broad-spectrum, multi-class analytical techniques.

### 4.3. Expanding Positive Lists: Driving High-Throughput MRMs and Sample Preparation

The volume of pesticides regulated under positive list systems presents a significant analytical challenge. Testing for hundreds of potential residues on a single sample, such as an apple or maize grain, would be economically and logistically unfeasible using traditional single-residue methods. This regulatory reality has been a primary catalyst for the development and widespread adoption of Multi-Residue Methods (MRMs). The quantitative expansion of target compound coverage in multi-residue methods (MRMs) directly mirrors the rapid growth of global regulatory positive lists. Historically, analytical methods were restricted to single-residue or narrow-class analyses. For instance, early liquid chromatography-tandem mass spectrometry (LC-MS/MS) protocols accommodated only one to three specific antiviral drugs per run due to the divergent physicochemical properties of the analytes [59]. To minimize analytical costs and increase operational efficiency [18], instrumental development scales with regulatory demands. Modern confirmatory analysis has transitioned to multi-class configurations that multiplex hundreds of compounds within a single analytical method [46].

Currently, official European control laboratories deploy MRMs covering over 300 pesticides [60]. Furthermore, integrated methodologies combining LC-MS/MS and gas chromatography-tandem mass spectrometry (GC-MS/MS) operating in selected reaction monitoring modes routinely facilitate the concurrent screening of up to 551 pesticides in a single workflow [14]. Similarly, contemporary targeted MS/MS protocols achieve the simultaneous detection of 100 distinct registered and banned veterinary drugs across highly complex biological matrices [61]. Ultimately, this continuous escalation in instrumental scanning speed and data acquisition capacity corresponds to a necessary hardware adaptation. Laboratories are required to implement these high-throughput platforms to analyze extensive and heterogeneous international regulatory lists within strict financial and temporal constraints.

To match this instrumental throughput, the analytical bottleneck shifts to sample preparation. Under the pressure of expanding global MRL monitoring systems, the evolution of sample preparation is driven primarily by the operational necessity to maximize analytical throughput while simultaneously minimizing the cost-per-analysis and organic solvent consumption. The widespread adoption of the QuEChERS (Quick, Easy, Cheap, Effective, Rugged, and Safe) methodology [62] effectively replaces traditional liquid–liquid extraction (LLE) and standard solid-phase extraction (SPE) protocols, which historically consumed large volumes of expensive and environmentally harmful organic solvents [63]. Functionally, the QuEChERS protocol utilizes acetonitrile extraction followed by salt-induced phase partitioning to separate analytes from the aqueous matrix.

Because modern “positive lists” mandate the concurrent screening of hundreds of compounds with divergent chemical stabilities, the methodology incorporates specific buffer systems, primarily utilizing acetate or citrate salts, to stabilize pH-sensitive and degradable pesticides during extraction [64]. Furthermore, because international validation guidelines demand strict recovery rates regardless of matrix complexity, laboratories are required to adapt their clean-up strategies. To maintain strict regulatory compliance when facing increasingly complex food matrices, laboratories routinely modify the subsequent dispersive solid-phase extraction (d-SPE) clean-up step. The incorporation of advanced sorbents, such as multi-walled carbon nanotubes (MWCNTs) and graphitized carbon black (GCB) [14,65], functions as a necessary methodological adaptation to mitigate severe ion suppression and ensure validated recoveries.

Similarly, statutory Residue Definitions (RDs) that explicitly include highly polar, non-partitioning compounds dictate further methodological divergence. For these highly polar analytes, specialized procedural adaptations like the Quick Polar Pesticides (QuPPe) method bypass traditional partitioning steps entirely to retain target compounds. Beyond analyte recovery, overarching environmental and occupational safety regulations directly reshape laboratory workflows. Concurrently, green chemistry initiatives within official control laboratories strictly limit the use of highly toxic halogenated solvents, explicitly rejecting methods that rely on reagents like dichloromethane for sample partitioning [66]. This regulatory restriction necessitates the miniaturization of extraction protocols. Modernized methods proportionally scale down sample masses and solvent volumes from tens of milliliters to merely a few milliliters (e.g., from 20 mL to 4 mL) to integrate with high-throughput automated homogenization equipment [59]. Additionally, the enhanced sensitivity of contemporary mass spectrometers facilitates direct “dilute-and-shoot” approaches or the application of biodegradable deep eutectic solvents, which further minimizes solvent reliance [37]. Ultimately, these sample preparation innovations streamline laboratory workflows. They allow high-throughput facilities to consistently satisfy strict recovery and reproducibility criteria while reducing chemical waste disposal costs and manual labor, thereby sustaining the economic viability of routine compliance testing [67].

However, this drive for a universal, high-throughput extraction protocol frequently conflicts with specific regulatory mandates. Beyond the volume of target compounds, regulations often define the residue (Residue Definition, RD) not just as the parent pesticide but as the sum of the parent compound and its toxicologically relevant metabolites [68]. This adds a layer of analytical complexity, as the divergent physicochemical properties between a parent compound (often lipophilic) and its metabolites (often highly polar) frequently preclude extraction via a single MRM protocol. Consequently, laboratories are required to validate parallel, methodologically distinct workflows for a single target to ensure the calculated sum complies with the statutory RD.

### 4.4. The “Zero Tolerance” Imperative: MRPLs and the Push for Ultra-Trace Analysis

While many veterinary drugs are approved for use with established MRLs, a specific class of substances is strictly prohibited in food-producing animals due to severe human health risks, such as carcinogenicity or aplastic anemia [3,47]. These include compounds like chloramphenicol, nitrofurans, and malachite green, which are banned under EU, United States, and Chinese regulations [12,47].

For these banned substances, the regulatory policy is one of “zero tolerance.” In analytical terms, this translates to a requirement for “non-detection.” However, since absolute zero is an analytical impossibility, this policy mandates the use of ultra-trace analysis methods capable of reaching the lowest scientifically defensible LOD. The EU formalizes this concept with the establishment of Minimum Required Performance Limits (MRPLs), which have now evolved into Reference Points for Action (RPAs) under Regulation (EU) 2019/1871, establishing explicit analytical thresholds that laboratories must achieve to monitor for these banned compounds [47]. This regulatory pressure for continually lower detection limits drives the development of analytical instrumentation, often necessitating the use of high-sensitivity LC-MS/MS or High-Resolution Mass Spectrometry (HRMS) to achieve the required performance [69]. The detection of these substances at any level constitutes a violation, making the sensitivity of the analytical method a primary determinant of compliance.

### 4.5. The Matrix Reality: Validation Bottlenecks in Ultra-Trace Validation

While regulatory limits dictate the required analytical sensitivity, achieving these exact thresholds is frequently obstructed by the complexity of the sample matrix. Regulations govern an array of diverse agricultural commodities, presenting analytical challenges centered on matrix complexity and method validation [70]. MRLs and MLs are enforced on complex matrices where target analytes—ranging from pesticides to environmental contaminants like heavy metals and mycotoxins—are prone to accumulation. For example, recognizing that rice is a primary source of dietary cadmium exposure for its population, China has established a strict ML for cadmium in rice. Similarly, regulatory limits exist for lead in preserved eggs in China, mycotoxins in cereals and nuts across the EU and Codex, and pesticides like bifenthrin in tea, a notoriously difficult matrix [5,70].

The analytical implication of this matrix diversity is twofold. First, while streamlined extraction protocols (like QuEChERS) succeed for high-water, simple matrices, the inherent matrix complexity of high-value agricultural commodities (e.g., tea, spices, and dairy) significantly restricts their general applicability in global trade. During chromatographic separation and mass spectrometric analysis, complex food matrices—such as high-lipid animal products (e.g., camel milk), high-pigment fruits, and challenging botanical matrices like tea, hops, and spices—introduce co-extractive interferences [71]. These endogenous components frequently co-elute with target analytes, causing significant ion suppression or enhancement within the electrospray ionization (ESI) source [55,59]. For instance, high concentrations of natural pigments in vegetables and the complex protein-lipid matrix of dairy products directly interfere with precise quantification, leading to biased residue results and high rates of false positives or negatives [15]. To mitigate these matrix effects and ensure accurate quantification at stringent international regulatory limits, laboratories are required to implement matrix-specific optimization. While the addition of stable isotope-labeled internal standards (SIL-IS) for each target compound represents the optimal solution, the economic cost and limited availability of SIL-IS for hundreds of concurrently monitored analytes render this approach impractical for routine MRM applications [59]. Consequently, laboratories must adopt labor-intensive alternative strategies. The most prevalent approach necessitates the construction of matrix-matched calibration curves, wherein calibration standards are prepared using extracts of the specific blank commodity to compensate for ionization interferences [63,65]. Furthermore, extensive sample pre-treatment and dedicated clean-up procedures, such as precipitation techniques or advanced solid-phase extraction, are often mandatory prior to instrumental analysis [61]. Ultimately, the development of these customized, matrix-dependent compensation strategies increases the operational costs, analytical complexity, and turnaround times for laboratories attempting to secure global regulatory compliance for complex agricultural exports.

Second, strict regulatory compliance extends beyond instrumental capacity to encompass mathematically defined method validation protocols. Regulatory bodies frequently mandate highly specific operational instructions—ranging from defining residues strictly on a dry weight basis to requiring the use of advanced instrumentation like LC-MS/MS for veterinary drugs. Building upon these technical mandates, compliance with ISO/IEC 17025 and specific validation frameworks (e.g., SANTE) provides the necessary evidentiary basis for legal validity in international food trade. These frameworks serve as a standardized system that establishes quantitative criteria for recovery and precision (further detailed in Section 5.4) [13,68]. Furthermore, instrumental confirmation necessitates a signal-to-noise (S/N) ratio exceeding 10, alongside relative abundance-dependent dynamic tolerance windows for product ions mandated by modern guidelines (e.g., SANTE and EU 2021/808) [55,59]. However, applying these criteria to trace multi-residue analysis at extreme regulatory thresholds—such as default MRLs of 0.01 mg/kg—challenges the detection limits of current analytical instrumentation. At these trace concentrations, inherent baseline noise and co-extracted matrix components significantly amplify analytical variance, compromising overall measurement precision [70,72]. For instance, low-intensity interfering peaks in complex matrices frequently cause product-to-precursor ion ratios to exceed the allowable ±30% deviation, which forces legally unconfirmed results or regulatory false-negatives (due to failure to meet strict identification criteria) despite the actual presence of the target analyte [59,61]. Additionally, variable extraction efficiencies and compound instability across heterogeneous matrices limit the ability to formulate a rigorous, metrologically and statistically valid calculation of measurement uncertainty for hundreds of analytes simultaneously [60]. Ultimately, imposing static validation thresholds onto ultra-trace analytical environments increases the quality control burden for routine compliance laboratories. A systematic comparison of how these distinct regulatory logics—ranging from the positive list system to zero-tolerance RPAs—dictate specific analytical strategies and challenges is synthesized in Table 3.

### 4.6. Global Compliance Benchmarks and Regulatory Asymmetry

#### 4.6.1. The 0.01 mg/kg Default Limit: Regional Mandates as Global Performance Baselines

As introduced in Section 4.1., the enforcement of a 0.01 mg/kg default limit by regions such as the EU and Japan functions as a primary operational baseline for modern analytical instrumentation [2]. Both the EU and Japan have established this uniform “catch-all” limit (0.01 ppm) for any pesticide residue for which a specific MRL has not been set under their positive list systems. Furthermore, this exact low limit is specifically applied under the precautionary principle to foods intended for infants and young children.

Rather than a formal analytical capability metric (like the RPAs used for banned veterinary drugs), this statutory threshold dictates compliance requirements. To demonstrate compliance for export to these specific markets, a laboratory’s analytical method must possess a validated LOQ at or below 0.01 mg/kg. This stringent regional requirement has rendered many older detector technologies obsolete for regulatory pesticide screening and has been a major driver for the global adoption of highly sensitive tandem mass spectrometry (LC-MS/MS and GC-MS/MS), establishing it as a key performance benchmark for the analytical community.

#### 4.6.2. Asynchronous Standards and Defensive Technological Redundancy

**Figure 3:**
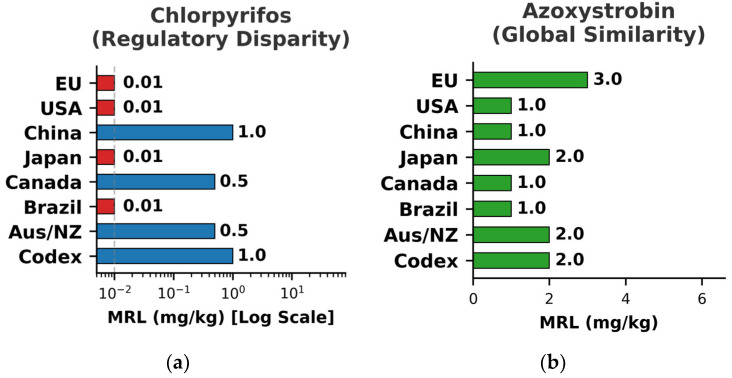
(**a**) Regulatory disparity in Maximum Residue Limits (MRLs) for chlorpyrifos on grapes across eight representative jurisdictions. The logarithmic scale highlights the orders-of-magnitude difference between markets enforcing strict default limits (e.g., EU, 0.01 mg/kg) or targeted tolerance revocations (e.g., United States) and those maintaining higher risk-based limits (e.g., China, Codex, 1.0 mg/kg). Data sources: [73,74,75,76,77,78,79,80]. (**b**) Global harmonization in MRLs for azoxystrobin on grapes. The linear scale demonstrates a high degree of consensus among major regulatory bodies, with limits clustering consistently between 1.0 and 3.0 mg/kg, reflecting a more unified approach to risk assessment for newer chemistries. Data sources: [73,74,75,76,77,78,79,80].

However, the global application of such stringent baselines is highly asymmetric. While international bodies like the Codex Alimentarius Commission (CAC) work to establish global standards, significant disparities in MRLs persist across different regulatory regions [3,18]. These differences have direct consequences for the operational scope of analytical laboratories serving the international food trade. For a single pesticide-commodity pair, MRLs can vary by orders of magnitude. For example, legacy MRLs for compounds like carbaryl or captan can vary by orders of magnitude between the United States and the EU. To systematically visualize this complex landscape, we compare two representative evolutionary scenarios in Figure 3. Figure 3a visualizes the significant regulatory disparity for chlorpyrifos, an older pesticide where MRLs vary widely due to differing national risk assessments and bans. In this case, the European Union enforces a strict default limit of 0.01 mg/kg, whereas China and Codex maintain limits up to 100 times higher (1.0 mg/kg). Conversely, Figure 3b depicts the emerging trend of harmonization for azoxystrobin, a modern fungicide where international standards show a high degree of consensus.

The juxtaposition of these two figures underscores the dual reality of the current regulatory environment. While harmonization is progressing for newer substances (Figure 3b), the fragmentation for legacy compounds (Figure 3a) remains a critical hurdle. To clear a product for global export, analytical laboratories are required to adopt a “lowest common denominator” strategy, validating methods to meet the strictest MRL of any potential destination market (typically 0.01 mg/kg) rather than the international average. This operational reality drives export-oriented laboratories into a costly posture of defensive technological redundancy—investing in ultra-high-sensitivity instrumentation and maintaining a complex, flexible portfolio of accredited methods to mitigate the compliance risks of asymmetric global standards.

In summary, this section establishes that food safety regulations function as primary drivers for analytical methodology. The paradigm shift toward expansive positive lists and zero-tolerance policies dictates the adoption of chromatography coupled with tandem mass spectrometry (e.g., LC-MS/MS and GC-MS/MS). However, an evaluation of the eight jurisdictions exposes a distinct regulatory paradox: while the analytical execution has achieved striking global homogeneity—anchored by MS/MS platforms and ISO 17025 validation—the regulatory targets remain fractured by the divide between hazard- and risk-based approaches. From a macro-analytical perspective, this persistent asymmetry transforms the extreme sensitivity of modern instrumentation into a “technological tax” paid by export-oriented economies to reconcile geopolitical regulatory divergence. Critically, this observation indicates that the current targeted monitoring paradigm is reaching a strategic and economic ceiling, where the unsustainable cost of maintaining these fragmented compliance capabilities now serves as the primary driver for a shift in analytical strategy. This reality underscores the necessity of moving beyond the relentless escalation of targeted sensitivities toward the integration of High-Resolution Mass Spectrometry (HRMS) and international data harmonization. By establishing how these regulatory thresholds dictate the current analytical execution and its inherent economic limits, the subsequent sections will trace these limits back to their toxicological origins, examining how risk assessment protocols generate the MRLs that drive these expanding analytical requirements.

## 5. Risk Assessment: The Toxicological and Dietary Origins of Analytical Limits

While the preceding sections establish that regulatory statutes function as the basis for chemical analysis—driving both the homogeneity and heterogeneity of global testing methodologies—these legal mandates are the administrative outcomes of scientific risk assessment. To understand why these regulations achieve convergence in some areas while remaining fragmented in others, it is essential to look beyond the legislative text and examine the underlying risk assessment process. This section explores how the internationally recognized risk analysis paradigm shapes the regulatory landscape.

### 5.1. The Toxicological Foundation: ADI and ARfD

The foundation of chemical risk assessment is hazard characterization, which establishes Health-Based Guidance Values (HBGVs) derived from toxicological data [81]. These values represent an estimate of the amount of a substance that a person can be exposed to daily over a lifetime without appreciable health risk. Two key HBGVs form the basis of regulatory limits for pesticides and contaminants: Acceptable Daily Intake (ADI): This value addresses chronic (long-term) exposure. It serves as the primary benchmark for setting limits on residues that may be consumed regularly over a lifetime [58]. Acute Reference Dose (ARfD): This value addresses acute (short-term) risk from exposure to a chemical over a brief period, typically a single meal or one day. It is particularly relevant for substances capable of causing immediate toxic effects [50].

These toxicological endpoints are determined through rigorous scientific evaluation, often by specialized bodies such as the European Food Safety Authority (EFSA) or Japan’s Food Safety Commission, and typically incorporate uncertainty factors to account for inter- and intraspecies differences.

### 5.2. The Role of Dietary Exposure

Once the ADI and ARfD are established, the next step in risk assessment is to estimate the potential dietary exposure of the population. While the Codex Alimentarius provides a foundational global benchmark for food safety, the uniform application of its Maximum Residue Limits (MRLs) across all jurisdictions is scientifically inappropriate due to variations in global food consumption patterns [22]. Within the internationally agreed risk assessment framework, regulatory limits are not derived solely from the intrinsic toxicity of a chemical; rather, risk is characterized by computing total dietary exposure, which necessitates multiplying the residue concentration by the specific food consumption data of a target population [81]. Consequently, localized dietary structures dictate exposure scenarios. For instance, rice consumption in China constitutes approximately 50% of the total dietary exposure to cadmium, creating a distinct exposure pathway compared to Western diets [8]. Similarly, specific demographic groups, such as Dutch toddlers consuming maize-based products or South Asian populations consuming wheat-based chapattis, exhibit dietary intakes of specific commodities that are multiple times higher than those of other populations [48]. If an MRL is standardized globally without accounting for these high-frequency, high-volume consumption habits, the resulting localized exposure can easily exceed established Health-Based Guidance Values (HBGVs), such as the Acceptable Daily Intake (ADI) or Acute Reference Dose (ARfD) [82].

Recognizing this variable, national regulatory authorities regularly deviate from Codex recommendations to fulfill their statutory obligation to protect vulnerable domestic populations [50]. For example, the European Food Safety Authority (EFSA) frequently rejects proposed Codex MRLs (CXLs) for specific crops—such as apples or certain animal commodities—when its localized Pesticide Residues Intake Model (PRIMo) identifies acute exposure concerns for European consumers [56]. Likewise, under World Trade Organization (WTO) agreements, China legitimately establishes stricter-than-Codex limits for cadmium in rice to mitigate its specific national public health risk [8]. A proposed regulatory limit is legally enforceable only if the mathematical calculation of local exposure—utilizing residue data from supervised field trials—proves that it does not exceed these health-based guidance values [55]. These field trials provide the Supervised Trial Median Residue (STMR) for chronic exposure assessment and the Highest Residue (HR) for acute exposure assessment [52]. Because the consumption variable (the denominator in safety equations) is determined by cultural and regional habits, the resulting MRLs must diverge to maintain equivalent toxicological protection. This variation implies that MRL fragmentation is not a mere regulatory byproduct but a mathematical necessity of geographic dietary diversity.

### 5.3. From MRL to LOQ: Deriving the Analytical Requirement

The final output of the risk management process is the Maximum Residue Limit (MRL), the highest level of a pesticide or contaminant residue that is legally tolerated in or on a food commodity [82]. The MRL is set at a level that is both safe for consumers (i.e., ensures dietary exposure is below the ADI/ARfD) and achievable under Good Agricultural Practice (GAP) [58].

For the analytical laboratory, the MRL serves as the primary performance target. To enforce an MRL, the analytical method used for monitoring and control must be capable of reliably quantifying the chemical at or below that legal limit. This requirement translates the MRL into a mandatory Limit of Quantification (LOQ) [68]. The relationship is explicit: LOQ ≤ MRL.

As established in the preceding chapters, this mathematical mapping is most evident under the default 0.01 mg/kg mandates. Under such frameworks, limits set at the analytical detection boundary are frequently marked with an asterisk (*), making the translation from legal limit to mandatory instrumental LOQ unambiguous [68].

### 5.4. Method Validation: Ensuring Data Reliability and Comparability

To ensure that analytical results are accurate and comparable across laboratories and jurisdictions, regulatory frameworks mandate that methods used for official controls be thoroughly validated. This requirement is operationalized through the quality management frameworks established in Section 3.4 and detailed in specific regulatory guidance documents, most notably the EU’s SANTE/11312/2021 v2026 guidelines and the United States Food and Drug Administration (FDA) guidelines—whose scientific frameworks are grounded in analytical research, such as the empirical derivation of measurement uncertainty [83], the historical evolution of quality control [84], and core mass spectrometric validation principles [85]—which are referenced by analysts worldwide [63]. These guidelines define the performance characteristics that a chemical analysis method must meet, as summarized in Table 4. Key validation parameters include:

Accuracy (Recovery): The method must demonstrate its ability to accurately measure a known concentration of an analyte spiked into a food sample. While specific ranges can vary by analyte and matrix, guidelines like SANTE typically require mean recoveries in the range of 70–120% [63]. However, recognizing practical limitations, these thresholds are conditionally expanded (e.g., down to 50%) for complex matrices or sub-trace concentrations. For instance, one study validating a multi-residue method for rice reported acceptable recoveries between 58% and 104%, reflecting the permissible analytical tolerances required at trace concentration levels [55].

Precision: The method must produce consistent, reproducible results. The SANTE guidelines specify a clear performance criterion for precision, requiring a repeatability relative standard deviation (RSD) of ≤20% [63].

Linearity: The method must show a proportional relationship between the analytical signal and the concentration of the analyte over a defined range, confirming its sensitivity across different levels [63].

Limit of Quantification (LOQ): As the primary analytical requirement, the LOQ is defined as the lowest concentration of an analyte that can be quantitatively determined with acceptable accuracy and precision. For a method to be fit-for-purpose for regulatory monitoring, its validated LOQ must be at or below the MRL for the specific pesticide-commodity combination [63].

These validation requirements are not confined to one region. For instance, Brazil employs a national manual for analytical quality that aligns with EU SANCO guidelines [63], demonstrating a global consensus on the analytical rigor needed to support food safety regulation.

### 5.5. The Instrumental Imperative for Tandem Mass Spectrometry

Because modern risk assessments often utilize complex residue definitions (encompassing both lipophilic parents and polar metabolites), and validation guidelines demand high selectivity, conventional detectors are insufficient to satisfy regulatory mandates. Consequently, the operational reality of official control is linked to HPLC and GC coupled with tandem mass spectrometry (MS/MS) [64]. As discussed previously, these highly selective platforms function as the physical interface required to ensure that analytical outputs map reliably onto the strict exposure limits generated by toxicological risk assessments [61]. In this architecture, tandem mass spectrometry functions as the analytical mechanism capable of reconciling the tension between universal validation criteria and highly variable regional limits. By providing the necessary sensitivity to reach the ‘lowest common denominator’ of global standards, these instruments act as the primary technical mechanism for ensuring robust compliance across a fragmented regulatory landscape.

Ultimately, the synthesis of the risk assessment paradigm reveals a distinct metrological challenge in global food safety. As established in Section 5.1 and Section 5.4, the biological baselines (toxicological ADI/ARfD) and the mathematical validation parameters (e.g., ISO/SANTE recovery and precision criteria) have achieved near-universal harmonization. Yet, because localized dietary models (Section 5.2) mathematically dictate exposure limits, the final regulatory outputs (MRLs) remain fragmented. This leads to a clear deduction: the global harmonization of MRLs is not merely hindered by political inertia; it is constrained by the mathematical reality of diverse human consumption. Consequently, analytical laboratories cannot wait for a homogenized international regulatory framework that may never materialize. Instead, the analytical sector is required to manage this regulatory divergence at the laboratory level. Because regulatory limits cannot be standardized upward, the global analytical community is driven to standardize detection capabilities downward—anchoring onto the strictest default baselines (e.g., LOQ ≤ 0.01 mg/kg). Therefore, the widespread operational mandate for LC/GC-MS/MS is not simply a result of analytical evolution, but a pragmatic compliance strategy. These ultra-trace instrumental platforms have effectively become the essential technological infrastructure required to navigate persistent regulatory gaps, proving that in modern food trade, advanced analytical capabilities are the primary mechanism for managing legislative asymmetry.

## 6. Limitations of Current Frameworks and Future Analytical Trajectories

As the current regulatory paradigm pushes targeted analytical methods to their physical and economic limits, this section explores the resulting operational and environmental challenges faced by global laboratories (Section 6.1 and Section 6.2). In response to these operational constraints and the emergence of unregulated hazards, we outline the necessary paradigm shift toward high-resolution mass spectrometry and adaptable validation strategies as the future trajectories of food safety analysis (Section 6.3 and Section 6.4).

### 6.1. The Operational and Economic Burden of Regulatory Asymmetry

As established in Section 4, the global landscape of food safety is characterized by regulatory asymmetry, driven by diverging risk philosophies and dietary exposure profiles. While the conceptual shift towards a universal baseline (e.g., the 0.01 mg/kg default) dictates modern instrumental design, the real-world application of asynchronous Maximum Residue Limits (MRLs) across jurisdictions creates profound economic and operational consequences for analytical facilities. In practice, this regulatory heterogeneity results in high border rejection rates, as evidenced by frequent notifications in the EU Rapid Alert System for Food and Feed (RASFF) concerning pesticide residues or aflatoxin-contaminated commodities, the vast majority of which involve rapid rejections at the European Economic Area (EEA) border [26,36]. To mitigate the risk of costly shipment rejections, laboratories in export-oriented sectors cannot rely on their domestic, often less stringent, “fit-for-purpose” standards [2,3]. Instead, they are required to align their compliance frameworks to mirror the strictest global limits [16,91]. Ultimately, this geographic regulatory mismatch alters laboratory economics, transforming the pursuit of high analytical sensitivity from a purely scientific endeavor into a major financial bottleneck that affects emerging agricultural economies and constrains global supply chains [22,72].

This escalating compliance burden extends beyond financial costs; it impacts routine laboratory operations. Aligned with these stringent regulatory demands, the cornerstone of modern pesticide residue testing is the Multi-Residue Method (MRM), which utilizes LC-MS/MS to screen for hundreds of compounds in a single, efficient analytical run. However, although MRMs significantly elevate analytical throughput, standardizing regulatory compliance solely through MRMs remains impractical. MRMs utilizing standard QuEChERS protocols exhibit physicochemical boundaries. Highly polar, ionic, or rapidly degradable analytes—such as glyphosate, phosphonic acid, and dithiocarbamates—exhibit poor retention in generic reverse-phase chromatography and fail to partition effectively during multi-class extraction [62]. This chemical incompatibility restricts the analytical scope and necessitates the implementation of dedicated single-residue methods (SRMs). To ensure statutory compliance for these specific targets, laboratories must isolate them using specialized techniques, such as the Quick Polar Pesticides (QuPPe) method or modified colorimetric cleavage procedures [13,15]. Consequently, SRMs represent an essential component for comprehensive residue monitoring.

Furthermore, deploying standardized analytical methods across different geographical and operational environments at these stringent regulatory thresholds introduces analytical challenges. When a validated method transfers between facilities, its performance frequently faces the risk of deviating from original metrics due to “validation drift.” This drift is driven by specific engineering and environmental variables. For example, hardware configurations across liquid chromatography-tandem mass spectrometry (LC-MS/MS) platforms—where even identical instrument models from the same vendor require customized ion source temperatures and desolvation gas flows to meet baseline specifications—directly alter ionization efficiencies [72]. Concurrently, minor discrepancies in local solvent purity, variations in pre-analytical sample storage conditions, and inherent matrix inhomogeneity consistently hamper method reproducibility and amplify baseline noise [51]. Collaborative inter-laboratory studies confirm that these cumulative variations increase analytical variance. Such hardware and environmental discrepancies frequently push product ion ratios beyond strict regulatory tolerances, compromising data comparability and generating legally unconfirmed results or regulatory false-negatives [61].

### 6.2. The Environmental Footprint of Ultra-Trace Analysis

Beyond the operational and financial challenges inherent to these intensive compliance strategies, the continuous push for lower Maximum Residue Limits (MRLs) creates a growing tension with macro-ecological sustainability by mandating highly resource-intensive analytical practices. To achieve and sustain ultra-trace detection limits, official control laboratories operate advanced liquid chromatography-tandem mass spectrometry (LC-MS/MS) and high-resolution mass spectrometry (HRMS) platforms continuously. The physical operation of these instruments consumes substantial baseline energy, primarily driven by the mechanical roughing and turbomolecular pumps required to maintain ultra-high vacuum conditions, alongside the constant heating of high-purity nitrogen used as a desolvation gas in the ionization source. Furthermore, the extensive extraction and chromatographic separation processes required prior to instrumental analysis generate large volumes of toxic aqueous and organic waste. As noted earlier in the context of extraction miniaturization, routine compliance methods have historically relied heavily on hazardous reagents, such as dichloromethane for sample partitioning or carcinogenic preservatives like sodium azide, which generate occupational and ecological risks [66].

This substantial operational footprint necessitates the systematic application of Green Analytical Chemistry (GAC) principles to mitigate the adverse impacts of routine regulatory procedures on the environment. To evaluate this, modern greenness assessment tools, including the Analytical GREEnness (AGREE) metric and the Green Analytical Procedure Index (GAPI), quantitatively assess the ecological impact of entire analytical workflows. These standardized frameworks penalize methods that consume excessive electrical energy, emit hazardous liquid waste, or utilize highly toxic reagents, thereby evaluating the environmental cost required to achieve ultra-trace detection limits. Authorities must balance analytical sensitivity with sustainability by mandating greenness metrics during method validation, ensuring that the ecotoxicological cost of a technique does not offset the public health benefits it provides.

### 6.3. Transitioning from Targeted Monitoring to Non-Targeted Screening (HRMS)

Beyond the environmental and physical resource demands inherent to ultra-trace MRMs (discussed in Section 6.2), a separate limitation lies in analytical scope. While LC-MS/MS remains the primary standard for enforcing known regulatory limits (as established in Section 5.5), its limitation lies in its inability to detect anything outside the pre-defined target list. This limitation creates a gap in the food safety system, as conventional testing panels often exclude unexpected contaminants, adulterants, or by-products of novel processing techniques [92]. The 2008 melamine incident, where a nitrogen-rich industrial chemical was deliberately added to infant formula and pet food, demonstrated how targeted methods can fail to detect unanticipated, harmful substances [92].

**Figure 4:**
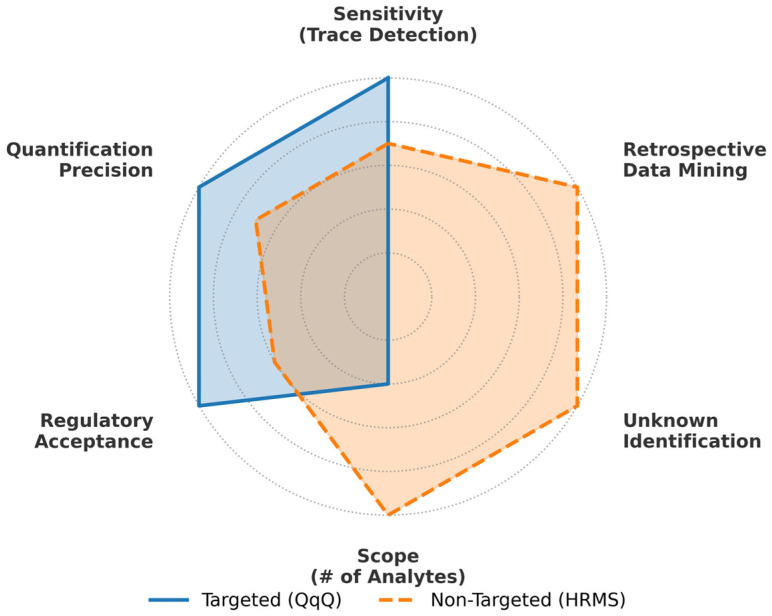
Comparative performance profile of Targeted Analysis (using Triple Quadrupole MS) versus Non-Targeted Screening (using High-Resolution MS). While targeted methods currently excel in sensitivity, precision, and regulatory acceptance (blue area), HRMS technologies offer superior capabilities for identifying unknown compounds and performing retrospective data mining (orange area), addressing the emerging need to detect food fraud and novel contaminants.

In response, regulations are evolving to address Economically Motivated Adulteration (EMA), as seen in the United States Food Safety Modernization Act (FSMA) [24]. This regulatory shift drives a change in analytical strategy, transitioning from purely targeted detection to non-targeted screening. This shift necessitates the adoption of High-Resolution Mass Spectrometry (HRMS) platforms, such as Quadrupole Time-of-Flight (Q-TOF) or Orbitrap systems. As noted in recent studies, “advances in untargeted metabolomics, foodomics, and chemometric profiling provide promising tools to identify anomalies that may indicate fraud, even in the absence of predefined target compounds” [92]. By capturing a full-spectrum dataset, HRMS allows for the retrospective identification of contaminants and the detection of atypical chemical profiles that may signal adulteration, even when the specific adulterant is unknown [69,93]. This progression beyond simply testing for a list of known contaminants is essential for the “early identification and possible prevention of food fraud instances” [22]. Figure 4 illustrates the comparative strengths and weaknesses of traditional targeted methods (MRM) versus emerging non-targeted screening (HRMS), highlighting the trade-off between sensitivity/precision and the ability to detect unknown hazards.

### 6.4. Analytical Challenges for Emerging Contaminants and Novel Foods

The food industry is characterized by rapid innovation, with the constant introduction of new ingredients, materials, and processes. While this drives economic growth, it also presents emerging food safety challenges for which regulatory and analytical frameworks are often unprepared. Contaminants such as nanoparticles from new packaging materials or ingredients used in novel processes like 3D food printing represent a new frontier for chemical analysis [22]. For many of these emerging hazards, there is a significant lack of established regulatory limits and a lack of standardized, validated analytical methods [30].

This situation places analytical laboratories in a challenging position, tasked with developing and validating methods “in-house” to detect and quantify substances for which there may be no official protocols, certified reference materials, or international proficiency testing schemes. This not only increases the cost and complexity of testing but also raises questions regarding the comparability and legal defensibility of the data produced. The rapid pace of technological development in food production suggests that any system reliant on a fixed list of “official” analytical methods risks becoming obsolete [22]. Addressing this challenge requires a more adaptable approach to method development and validation, supported by international collaboration among regulatory bodies and organizations like ISO and AOAC International to keep pace with innovation [10], ensuring that future analytical frameworks remain proactive rather than merely reactive to shifting global supply chains.

In summary, the reliance on targeted multi-residue methods—historically driven by the necessity to navigate a fragmented landscape of stringent, localized MRLs—has reached its operational, economic, and ecological limits. Consequently, continuously lowering detection limits for static lists of known compounds is no longer a viable long-term strategy. As demonstrated by the vulnerabilities to food fraud and the emergence of novel food matrices, addressing these challenges requires a shift in analytical methodology. The future of regulatory enforcement relies on transitioning from reactive, target-list quantification toward proactive, non-targeted chemical profiling using High-Resolution Mass Spectrometry (HRMS). Coupled with the integration of green chemistry principles and adaptable, internationally cooperative validation frameworks, this technological evolution provides a sustainable pathway for laboratories to safeguard an increasingly complex global food supply network.

## 7. Conclusions

This review has established that global food safety regulations function as much more than administrative boundaries; they serve as the technical specifications that dictate the evolution of analytical chemistry. While the global community has successfully harmonized the preventive philosophy of food safety and the quality assurance metrics (e.g., ISO/IEC 17025), the core targets of compliance—Maximum Residue Limits (MRLs)—remain fractured. As demonstrated, this fragmentation is not a political failure but a mathematical consequence driven by localized dietary exposure models. Consequently, analytical laboratories have been necessitated to maintain “defensive technological redundancy.” To navigate this asymmetry, the analytical sector relies on the high sensitivity of liquid chromatography-tandem mass spectrometry (LC-MS/MS), applying ultra-trace default limits (e.g., 0.01 mg/kg) as a technical baseline to manage regulatory discrepancies during analysis.

However, this review concludes that the current paradigm of targeted monitoring has reached its operational, economic, and ecological limits. The analytical community can no longer address regulatory asymmetry by continuously reducing Limits of Quantification (LOQs) for static, predefined lists of compounds. Therefore, the future trajectory of chemical analysis must transition from the quantification of the known to the screening of the unknown. The integration of High-Resolution Mass Spectrometry (HRMS) for non-targeted analysis, coupled with the adoption of green chemistry principles, represents a sustainable path. Ultimately, as global supply chains face emerging, unregulated hazards and economically motivated adulteration, regulatory authorities must move beyond enforcing specific thresholds. The future requires the co-development of adaptable, internationally recognized validation frameworks that empower analytical laboratories to act as contributors to global food integrity.

## Figures and Tables

**Table 1:** Top 10 global exporters and importers of food in 2022.

Top 10 Exporters	Export Value (Billion USD)	Top 10 Importers	Import Value (Billion USD)
**European Union**	**693**	**European Union**	**653**
**United States**	**185**	**China**	**224**
**Brazil**	**132**	**United States**	**220**
**China**	**88**	**Japan**	**80**
**Canada**	**71**	United Kingdom	74
Indonesia	58	**Canada ^(1)^**	**48**
Argentina	54	Korea, Republic of	44
India	50	Mexico ^(1)^	38
Mexico	48	India	34
**Australia**	**45**	Saudi Arabia, Kingdom of	28

Note: Data is sourced from the external Table A15 of the World Trade Statistical Review 2023 [20] published by the World Trade Organization (WTO). Bold text indicates the specific jurisdictions selected for detailed examination in this review. ^(1)^ Imports are valued f.o.b.

**Table 2:** Overview of the legislative frameworks governing food safety in key jurisdictions and their specific mandates for chemical analysis.

Jurisdiction & Agency	Key Statute/Regulation	Core Regulatory Approach	Implications for Chemical Analysis
United States (FDA)	Food Safety Modernization Act (FSMA, 2011) [25]	Preventive controls (HARPC); Risk-based verification.	Mandates testing by accredited laboratories (LAAF rule); Focus on environmental monitoring and allergen control.
European Union (EFSA)	Regulation (EC) No 178/2002 (General Food Law) [27]	“Farm-to-Fork” traceability; Precautionary Principle.	Requires high-sensitivity Multi-Residue Methods (MRMs); Stringent method validation per SANTE guidelines.
China (SAMR)	Food Safety Law (2015 Amendment) [28]	“Strictest supervision”; Integrated national standards.	Consolidation of analytical standards (GB Standards); Heavy penalties drive demand for rapid screening and confirmatory testing.
Canada (CFIA)	Safe Food for Canadians Act (SFCA, 2012) [31]	Outcome-based; Preventive control plans (PCPs).	Emphasis on traceability; Flexible method selection provided performance criteria (LOD/LOQ) are met.
Japan (MHLW)	Food Sanitation Law (2018 Amendment) [32]	Positive List System.	Default limit (0.01 ppm) for unlisted chemicals necessitates high-sensitivity screening (e.g., GC-MS/MS, LC-MS/MS).
Brazil (ANVISA)	Law No. 11.346; ANVISA Resolutions (RDC) [33]	Risk-based inspection; Equivalency with EU/US.	Focus on mycotoxins and pesticide residues in export commodities; Harmonization with Codex MRLs.
Australia & NZ (FSANZ)	Food Standards Code (FSANZ Act 1991) [34]	Bi-national standard setting; Risk analysis.	Shared analytical protocols; Focus on dietary exposure assessment and total diet studies.
Codex (Intl.)	General Principles of Food Hygiene (CXC 1-1969) [35]	Science-based risk analysis.	Establishes global benchmark MRLs; Defines criteria for method validation and sampling (CAC/GL 27).

Note: FDA: U.S. Food and Drug Administration; EFSA: European Food Safety Authority; SAMR: State Administration for Market Regulation (China); CFIA: Canadian Food Inspection Agency; MHLW: Ministry of Health, Labour and Welfare (Japan); ANVISA: Brazilian Health Regulatory Agency; FSANZ: Food Standards Australia New Zealand.

**Table 3:** Classification of analytical strategies and challenges based on the underlying regulatory logic of global food safety frameworks.

Regulatory Logic	Target Hazard Category	Key Control Metric	Primary Analytical Strategy	Critical Analytical Challenge
Positive List System (Substances allowed only if explicitly listed)	Pesticides; Approved Veterinary Drugs	MRLs (Default limit: 0.01 mg/kg)	Multi-Residue Methods (MRMs) (e.g., QuEChERS + LC-MS/MS)	High-throughput screening of hundreds of analytes; Managing “sum of residues” definitions (parent + metabolites).
Zero Tolerance (Substances strictly prohibited due to severe risk)	Banned Veterinary Drugs (e.g., Nitrofurans, Chloramphenicol)	RPA/MRPL (Reference Point for Action)	Ultra-Trace Analysis (Targeted, High-Sensitivity)	Achieving extremely low LODs (ppb/ppt level) to prove absence; Preventing false positives in trace analysis.
Risk-Based/ALARA (Contaminants managed to As Low As Reasonably Achievable)	Environmental Contaminants (Mycotoxins, Heavy Metals)	Maximum Levels (MLs) (Matrix-specific limits)	Matrix-Specific Methods (Rigorous extraction & cleanup)	Overcoming severe matrix interferences (e.g., Cadmium in rice); Complex validation (e.g., dry weight basis).

Note: MRL: Maximum Residue Limit; MRPL: Minimum Required Performance Limit; RPA: Reference Point for Action; LOD: Limit of Detection; ALARA: As Low As Reasonably Achievable principle used for unavoidable contaminants.

**Table 4:** Harmonized analytical method performance criteria for chemical residue analysis across major global regulatory frameworks.

Validation Parameter	Definition/Role	General Acceptance Criteria (Quantitative Methods)	Key Regulatory References
Accuracy (Trueness/Recovery)	The closeness of the experimental value to the true value, typically measured via spiking experiments.	Mean recovery: 70–120% (Expanded range 50–120% often acceptable for difficult matrices or levels < 10 μg/kg)	EU SANTE/11312/2021 v2026 [68] United States FDA ORA-LAB.5.10 [72] China GB/T 27404-2008 [86]
Precision (Repeatability)	The consistency of results under the same conditions (same analyst/instrument/short time).	RSD ≤ 20% (Stricter RSD ≤ 15% often required for higher concentrations)	EU SANTE/11312/2021 v2026 [68] Codex CAC/GL 40-1993 [87] Japan MHLW Notification [88]
Limit of Quantification (LOQ)	The lowest concentration that can be quantified with acceptable recovery and precision.	LOQ ≤ MRL (Ideally LOQ ≤ 0.5 × MRL to ensure compliance reliability)	EU Reg. 396/2005 [42] Brazil MAPA Manual [89] China GB 2763 [90]
Selectivity/Specificity	The ability to differentiate the analyte from matrix interferences (e.g., co-eluting peaks).	No interfering peaks > 30% of LOQ (At the retention time of the target analyte)	EU SANTE/11312/2021 v2026 [68] US FDA Bioanalytical Method Validation Guidance

Note: RSD: Relative Standard Deviation; MRL: Maximum Residue Limit; LOQ: Limit of Quantification. Criteria derived from quantitative method validation guidelines for pesticide residues.

## Data Availability

No new data were created or analyzed in this study. Data sharing is not applicable to this article.

## Abbreviations

The following abbreviations are used in this manuscript:

ADI	Acceptable Daily Intake
ALARA	As Low As Reasonably Achievable
ANVISA	Brazilian Health Regulatory Agency (Agência Nacional de Vigilância Sanitária)
ARfD	Acute Reference Dose
CAC	Codex Alimentarius Commission
CDC	Center for Disease Control and Prevention (China)
CFIA	Canadian Food Inspection Agency
CFSA	China National Center for Food Safety Risk Assessment
EFSA	European Food Safety Authority
EMA	Economically Motivated Adulteration
EPA	Environmental Protection Agency (USA)
EU	European Union
EURL	European Union Reference Laboratory
FDA	Food and Drug Administration (USA)
FSANZ	Food Standards Australia New Zealand
FSMA	Food Safety Modernization Act (USA)
GAP	Good Agricultural Practice
GC-MS/MS	Gas Chromatography-Tandem Mass Spectrometry
HBGV	Health-Based Guidance Value
HRMS	High-Resolution Mass Spectrometry
ICP-MS	Inductively Coupled Plasma Mass Spectrometry
ISO	International Organization for Standardization
LC-MS/MS	Liquid Chromatography-Tandem Mass Spectrometry
LOD	Limit of Detection
LOQ	Limit of Quantitation
MAPA	Ministry of Agriculture, Livestock and Food Supply (Brazil)
MHLW	Ministry of Health, Labour and Welfare (Japan)
ML	Maximum Level
MPL	Minimum Performance Limit
MRL	Maximum Residue Limit
MRM	Multi-Residue Method
MRPL	Minimum Required Performance Limit
NFSS	National Food Safety Standards (China)
NRL	National Reference Laboratory
PT	Proficiency Testing
Q-TOF	Quadrupole Time-of-Flight Mass Spectrometry
QuEChERS	Quick, Easy, Cheap, Effective, Rugged, and Safe (extraction method)
RSD	Relative Standard Deviation
SAMR	State Administration for Market Regulation (China)
SFCA	Safe Food for Canadians Act
SPS	Sanitary and Phytosanitary Agreement (WTO)
SRM	Single-Residue Method
STMR	Supervised Trial Median Residue
USDA	United States Department of Agriculture
WTO	World Trade Organization
